# The variability and seasonality of the environmental reservoir of *Mycobacterium bovis* shed by wild European badgers

**DOI:** 10.1038/srep12318

**Published:** 2015-08-06

**Authors:** Hayley C. King, Andrew Murphy, Phillip James, Emma Travis, David Porter, Yu-Jiun Hung, Jason Sawyer, Jennifer Cork, Richard J. Delahay, William Gaze, Orin Courtenay, Elizabeth M. Wellington

**Affiliations:** 1 University of Warwick, School of Life Sciences, Gibbet Hill Campus, Coventry, CV4 7AL; 2 Animal and Plant Health Agency, Weybridge, Woodham lane, New Haw, Addlestone, Surrey, KT15 3NB; 3 National Wildlife Management Centre, Animal and Plant Health Agency, Woodchester Park, Nympsfield, Gloucestershire, GL10 3UJ, UK; 4 European Centre for Environmental and Human Health, University of Exeter Medical School, Knowledge Spa, Royal Cornwall Hospital, Truro, Cornwall, TR1 3HD; 5 Warwick Infectious Disease Epidemiology Research (WIDER), University of Warwick, Coventry, CV4 7AL

## Abstract

The incidence of *Mycobacterium bovis*, the causative agent of bovine tuberculosis, has been increasing in UK cattle herds resulting in substantial economic losses. The European badger (*Meles meles*) is implicated as a wildlife reservoir of infection. One likely route of transmission to cattle is through exposure to infected badger urine and faeces. The relative importance of the environment in transmission remains unknown, in part due to the lack of information on the distribution and magnitude of environmental reservoirs. Here we identify potential infection hotspots in the badger population and quantify the heterogeneity in bacterial load; with infected badgers shedding between 1 × 10^3^ − 4 × 10^5^ *M. bovis* cells g^−1^ of faeces, creating a substantial and seasonally variable environmental reservoir. Our findings highlight the potential importance of monitoring environmental reservoirs of *M. bovis* which may constitute a component of disease spread that is currently overlooked and yet may be responsible for a proportion of transmission amongst badgers and onwards to cattle.

The incidence of *Mycobacterium bovis* in cattle herds in Great Britain (GB) has increased from 0.01% in 1979^1^ to 4.8% in 2012^2^. Control and compensation has cost the taxpayer £500 million over the past decade and this is predicted to increase to £1 billion over the next 10 years if further geographical spread is observed^3^; making bovine tuberculosis one of the most economically important animal health problems in the UK farming industry^4^.

The European badger is a wildlife reservoir involved in the transmission of *M. bovis* to cattle in the UK and RoI^5^,^6^. Once infected, badgers may intermittently shed *M. bovis* cells in sputum, faeces and urine^7^. One likely route of transmission to cattle is through exposure to infected badger urine and faeces. Although the location and extent of environmental *M. bovis* has not been previously quantified, indirect contact with infected faeces and urine may be an important exposure pathway^8^. *M. bovis* genomic DNA can survive outside the host for up to 21 months^9^ and cells have been shown to be viable by culture from mice fed soil in which *M. bovis* had been persisting for months^10^. The survival of shed *M. bovis* cells is likely to vary in space and time in relation to local environmental conditions and the distribution of infectious badgers. Understanding patterns in environmental contamination (defined as the presence of *M. bovis* genome equivalents in the environment) could aid in the design of more effective interventions, currently based on culling and vaccination strategies.

The availability of a method to quantify relative levels of environmental contamination with *M. bovis* could open up possibilities for monitoring spatial and temporal variation in risk and may help direct the implementation of disease control interventions. Currently the only means of measuring levels of infection in badger populations is through trapping and testing with BrockTB Stat Pak^**®**^(Stat Pak)^11^, Interferon gamma (IFNγ)^12^ and culture of clinical samples^13^. Cultivation, particularly from faecal material, has low sensitivity and is qualitative (Fig. 1). A qPCR method for non-invasive environmental monitoring of shedding was developed in our group^14^,^15^. This qPCR assay quantifies faecal shedding, a measure that correlates strongly with the level of infection within a social group as measured by immunoassay (Spearman’s rho = 0.92, p < 0.001)^16^. The only other non-invasive method for monitoring infection in badger populations is culture of faecal material, which is particularly insensitive (Fig. 1). Using this optimised qPCR assay we are able to report on the spatio-temporal reservoir of *M. bovis* from badger faecal shedding in a natural population over the course of a year. Badgers defecate in latrines within or at the edges of their territories^17^ and hence they can be used to identify a defined population of animals.

## Methods

### Sampling and Trapping

Fresh faecal samples were obtained from latrines associated with 12 badger social groups (Table 1) in Woodchester Park Gloucestershire through 2012 and 2013. Two intensive sampling periods of two weeks each were undertaken during the period of peak badger latrine activity in the spring and autumn of 2012 where up to 10 unique faecal samples were obtained from latrines associated with each social group on alternate days. Faecal samples were taken from latrines in closest proximity to the main sett of each social group. A second sampling regime was undertaken over a year long period where up to 10 unique fresh, faecal samples were taken from latrines associated with each social group per day over two non-consecutive days in each season, starting two days after trapping operations took place in that location. For the purpose of this study March—May was classified as spring, June—August as summer, September—November as autumn and December—February as winter.

Each of the 12 badger social groups in the study was trapped four times throughout the year, once per season, with variable numbers of animals trapped between groups and seasons (Table S1). Badgers were trapped using baited cage traps placed around the main setts of each social group and identified using a unique tattoo applied at the first capture of that animal. Trapped badgers from each of the 12 social groups were tested by BrockTB Stat-Pak^®^, IFNγ and culture of clinical samples. All experimental protocols were approved by the University of Warwick and the Food and Environment Research Agency Ethical Review Committee and carried out in accordance with the approved guidelines and under the license granted by the Home Office under the 1986 Animal (Scientific Procedures) Act.

### DNA Extraction and qPCR

Total community DNA was extracted from 0.1 g (+/−0.003 g) of faeces using the Fast DNA spin kit for soil (MP Biomedicals) following the manufacturer’s instructions. *M. bovis* was detected and quantified using a qPCR assay which targets the RD4 deletion region unique to the *M. bovis* genome (Specificity data Table S2). An initial qPCR screen of each sample was performed using an ABI 7500 Fast qPCR machine (ABI) with two technical replicates of each sample. Positive controls (8.5 × 10^5^ genome equivalents) and negative controls were also present in duplicate on each plate. PCR reactions were set up using 900 nM of each primer (RD4F ^5'^TGTGAATTCATACAAGCCGTAGTCG^3'^, RD4R ^5'^CCCGTAGCGTTACTGAGAAATTGC^3'^), 250 nM of Taqman probe (6FAM-AGCGCAACACTCTTGGAGTGGCCTAC—TMR), 1 mg ml^−1^ bovine serum albumen (BSA), 12.5 μl of Environmental Mastermix 2.0 (ABI), 10 μl of template and made up to 25 μl with molecular grade water (Sigma Aldrich). PCR cycling conditions were 50 °C for 2 min followed by 95 °C for 10 min then 40 cycles of 95 °C for 15 sec and 58 °C for 1 min. Samples exhibiting amplification in one or more technical replicates were taken on to full quantification using three technical replicates per sample under the same conditions. If one or more of the technical replicates of the quantification assay exhibited amplification the sample was deemed positive for *M. bovis*. Serial dilutions of *M. bovis* BCG Danish 1331 genomic DNA were used as standards for this quantification. An inhibition control assay previously described^14^ was used to detect the possibility of false negative results due to inhibition. Where significant inhibition was detected DNA was re-extracted from frozen aliquots and qPCR assays were repeated. The number of *M. bovis* genome equivalents was quantified independently by qPCR at The University of Warwick and APHA Weybridge (Supplementary Figure 1).

### Data Analysis

All data analysis was performed using the statistical program R. Logistic regression with social group (Old Oak) as the baseline was used to determine whether the number of positive samples varied amongst social groups throughout the year. Binomial generalised linear models (GLM) were performed to determine differences in *M. bovis* cells numbers shed between groups and between seasons. For spring two sampling days per social group were chosen to represent cross sectional sampling. Variability within groups was determined by calculating the median, upper and lower quartiles and range for each soil group.

The probability of detecting a false positive rate was 2%, calculated using known negative faecal samples obtained from captive badgers at APHA which were routinely tested for bTB using IFNγ. Negative samples were double blinded and randomly introduced into the experiment at both centres. The probability of detecting x false positive for a given number of samples was calculated using equation 1 where *p(x)* *=* the probability of exactly *x* false positives, *f* *=* the false positive rate, *n* *=* the number of samples and *x* *=* the number of false positives.


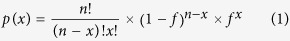


The number of confirmatory re-extractions (*e*) needed to result in the probability (*p*) of exactly *x* false positives was calculated using equation 2.


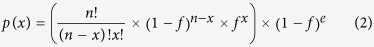


## Results

### Infection levels within social groups

**Figure 2:**
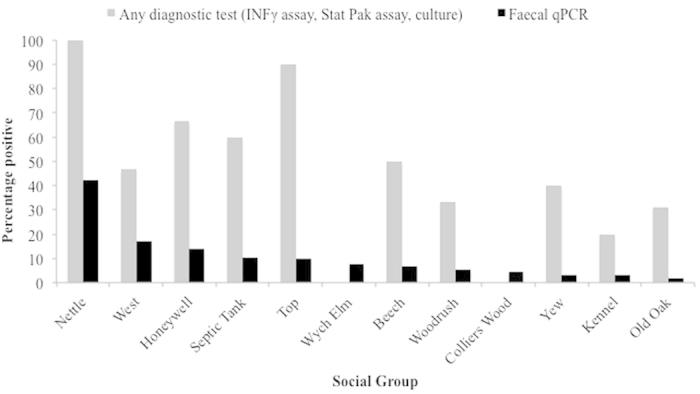
The percentage of badgers positive by any diagnostic tests compared to the percentage of positive faecal samples by qPCR per social group.

During the study, 53.6% of trapped badgers were *M. bovis* positive by Stat-Pak, IFNγ or culture. By qPCR faecal samples from every social group examined were found to be positive (Fig. 2). Although the percentage of infected faecal samples varied considerably (Table 1, Table S3), the numbers of *M. bovis* genome equivalents per faecal sample also varied widely ranged from 1 × 10^3^ to 4 × 10^5^ per gram of faeces (Table 1).

**Figure 3:**
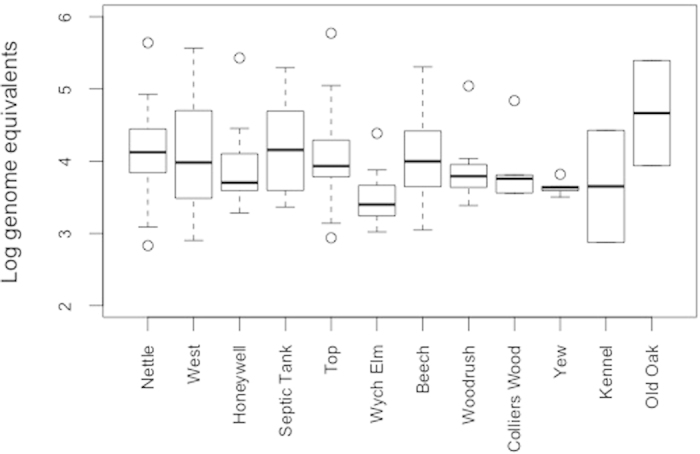
Distribution of *M. bovis* genome equivalents in positive samples by social group.

Significant variability in genome equivalents was identified both within and between social groups (Fig. 3) with social groups Nettle, Top, Septic Tank and West shedding more cells over the year than the other social groups (Table 1). Social groups with a high percentage of positive samples consistently shed amongst the highest cumulative numbers of *M. bovis* cells during the year (Table 1). Social group Old Oak was exceptional as it has one of the highest cumulative *M. bovis* genome equivalent values yet had the lowest percentage of positive samples in the study (Table 1). This distribution is consistent with the presence of a relatively small number of animals shedding large amounts of bacteria in some groups. However, as we could not assign faecal samples to individuals we cannot discount within-individual variation in shedding from being responsible for this observation. Hence the need for further research into heterogeneity in transmission risks amongst individual badgers.

### Seasonal variability in *M. bovis* shedding

**Figure 4:**
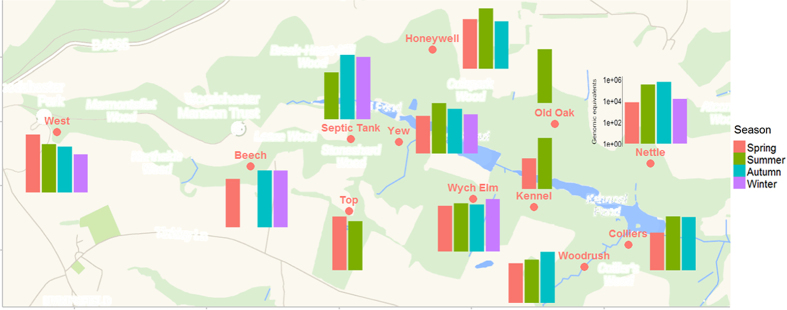
The cumulative *M. bovis* genome equivalents shed by each social group per season. Created in R version 3.0.2 using the packages ggplots 2^31^ and ggmaps^32^. The scales for all graphs are identical.

Overall a significantly greater number of *M. bovis* genome equivalents were shed in summer than in any other season. There were substantial seasonal differences in the cumulative number of *M. bovis* equivalents detected per social group (Fig. 4) with different groups identified as the largest contributors to the environmental pool of *M. bovis* throughout the year. Although summer had the highest number of genome equivalents overall, Septic Tank shed fewer cells in summer compared to other seasons and Top and shed more cells in spring. Nettle also shed fewer *M. bovis* genome equivalents in spring compared with the rest of the year. However, five social groups (Nettle, West, Honeywell, Septic Tank, and Top) were identified as having consistently high proportions of positive faeces and relatively large quantities of *M. bovis* bacilli shed (Table 1). This corresponds to immunoassay tests carried out on trapped badgers, which also identified these five groups as the most heavily infected (Table 1). Although there is strong correspondence between immunoassay and qPCR results there are some discrepancies, in particular Nettle and Top are 100% and 90% positive by immunoassay yet there was a large difference in the percentage of positive faecal samples with 42.2% and 10.0% respectively.

## Discussion

Detection of *M. bovis* by qPCR allows the presence of faecal shedding and hence infectious badgers to be established non invasively and raises the possibility of identifying infectious social groups. Unlike standard diagnostic tests the qPCR approach also quantifies levels of *M. bovis* shedding, providing opportunities to assess spatio-temporal variations in the environmental distribution of this potential source of infection for cattle, badgers and other wild mammals. Environmental transmission is likely to be a complex mixture of a number of factors including the infectious load of *M. bovis* in faeces and urine and changes in these reservoirs over time, proximity to cattle pasture, the frequency and type of contact cattle have with badger excrement and the age of faecal samples. The application of qPCR to further understand the epidemiology and transmission dynamics of bovine tuberculosis may be an important component in managing the advancing frontier between endemic and non-endemic cattle infection, and to inform transmission models (e.g. Brooks-Pollock *et al.* (2014)).

The heterogeneities observed in this study between social groups and the consistency with which five groups were identified as highly infected and shedding, suggesting that interventions targeted at particular high risk populations could have a larger impact than random and blanket control strategies^18^. However, we are mindful that any perturbation of badger populations could result in increased rather than decreased transmission^19^,^20^. The observed discrepancies in the percentage of positive faecal samples for social groups with similar prevalences of infection by immunoassay highlights the need for further work to establish the causes of these differences. Whilst heterogeneity in transmission is a well-known phenomenon, this study is one of the few empirical studies which have attempted to demonstrate the extent of this variability^21^. Although this study does not assess the viability of *M. bovis* in faeces, previous work has identified the presence of *M. bovis* 16S rRNA in soil^9^ and badger setts and latrines^22^. In addition, studies have had a culture success rate of 2.5% from badger faecal samples^23^ and *M. bovis* has been cultured from cattle faeces several months after excretion^24^. This indicates that at least a proportion of *M. bovis* cells shed in badger faeces can remain viable in the environment; however, further research is required to determine potential survival and transmissibility of *M. bovis* in environmental samples.

Whilst the focus in the UK and RoI is on badgers, other wildlife hosts are present^25^,^26^; however, little is currently known of their contribution to environmental reservoirs and their relative importance for transmission to cattle^25^. Issues controlling *M. bovis* are not confined to the UK and RoI. Worldwide there are problems with *M. bovis* in buffalo and lions in South Africa^27^, possums in New Zealand^28^, white tailed deer in America^29^ and wild boar in Spain^30^. This non-invasive qPCR assay can be employed to detect shedding in other systems and samples types including milk, water and clinical tissues, is possible using this method. Whilst controlling and monitoring *M. bovis* in wildlife populations remains a challenge, non-invasive monitoring of environmental contamination may open up opportunities to identify spatio-temporal heterogeneity in disease risks and hence contribute to the development of suitable approaches fro disease control in livestock.

## Additional Information

**How to cite this article**: King, H. C. *et al.* The variability and seasonality of the environmental reservoir of *Mycobacterium bovis* shed by wild European badgers. *Sci. Rep.* **5**, 12318; doi: 10.1038/srep12318 (2015).

## Supplementary Material

Supplementary Information

## Figures and Tables

**Figure 1:**
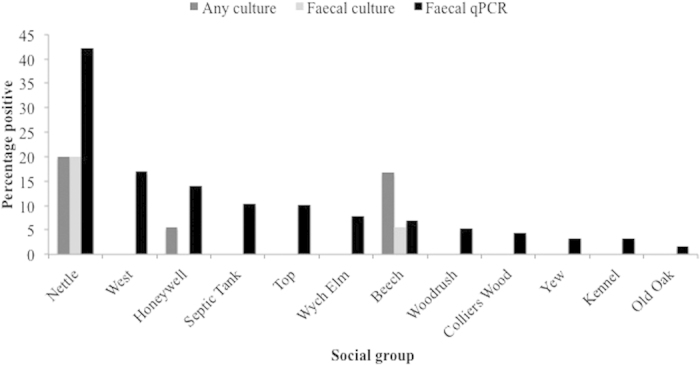
Percentage positive badgers per social group determined by any culture positive (tracheal or faecal) or faecal culture compared with positives by faecal qPCR. Data aggregated across the entire year.

**Table 1:** Summary of *M. bovis* genome equivalents counts by social group from faecal field sampling and immunoassay testing results on trapped badgers.

Social group	Percentage positive faeces by qPCR	Cumulative genome equivalents in faeces	Percentage positive IFNγ	Percentage positive Stat Pak	Percentage positive IFNγ or Stat Pak
Nettle	42.2	1.08 × 10^6^	60	100	100
West	16.9	1.48 × 10^6^	33.3	20	40
Honeywell	13.9	4.08 × 10^5^	50	50	66.7
Septic Tank	10.3	4.57 × 10^5^	40	20	60
Top	10.1	9.00 × 10^5^	20	90	90
Wych Elm	7.8	4.19 × 10^4^	0	0	0
Beech	6.9	4.98 × 10^5^	41.2	29.4	44.4
Woodrush	5.3	1.45 × 10^5^	0	33.3	33.3
Colliers Wood	4.3	8.83 × 10^4^	0	0	0
Yew	3.3	2.25 × 10^4^	0	40	40
Kennel	3.2	2.76 × 10^4^	0	20	20
Old Oak	1.6	2.56 × 10^5^	0	31.3	31.3
