# Supplementary material for: The variability and seasonality of the environmental reservoir of Mycobacterium bovis shed by wild European badgers
Source: Sci Rep. 2015 Aug 6;5:12318. doi: 10.1038/srep12318 (PMC4527091; doi:10.1038/srep12318)
Supplement: Supplementary Information [file srep12318-s1.pdf]

Supplementary information for:

The variability and seasonality of the environmental reservoir of *Mycobacterium bovis* shed by wild European badgers

Hayley C. King[1], Andrew Murphy[1], Phillip James[1], Emma Travis[1], David Porter[1], Yu-Jiun Hung[1], Jason Sawyer[2], Jennifer Cork[2], Richard J. Delahay[3], William Gaze[4], Orin Courtenay[1,5], Elizabeth M. Wellington[1]

[1] University of Warwick, School of Life Sciences, Gibbet Hill Campus, Coventry, CV4 7AL

[2] Animal and Plant Health Agency, Weybridge, Woodham lane, New Haw, Addlestone, Surrey, KT15 3NB

[3] National Wildlife Management Centre, Animal and Plant Health Agency, Woodchester Park, Nympsfield, Gloucestershire, GL10 3UJ, UK

[4] European Centre for Environmental and Human Health, University of Exeter Medical School, Knowledge Spa, Royal Cornwall Hospital, Truro, Cornwall, TR1 3HD

[5] Warwick Infectious Disease Epidemiology Research (WIDER), University of Warwick, Coventry, CV4 7AL

Corresponding Author: Hayley C. King, Hayley.King@Warwick.ac.uk, University of Warwick, School of Life Sciences, Gibbet Hill Campus, Coventry, CV4 7AL

Supplementary Figure One:

The correspondence between faecal qPCR testing at The University of Warwick and APHA Weybridge. Spearman's rho = 0.71, p = 0.01.

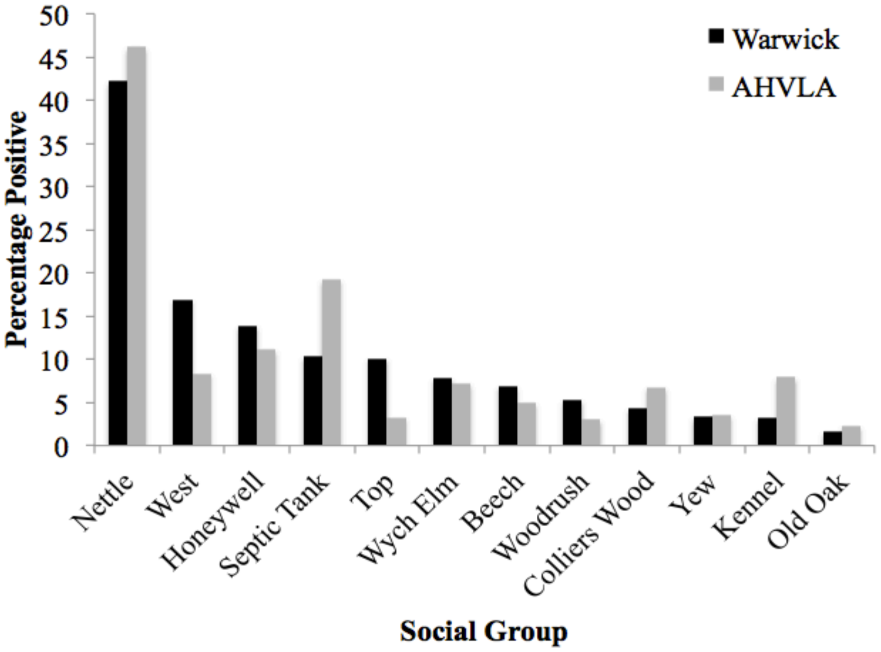

Table S1

The number of badgers trapped per social group, per season.

Social group	Spring	Summer	Autumn	Winter	Total
**Beech**	8	5	5	0	**18**
**Colliers Wood**	0	6	0	1	**6**
**Honeywell**	0	14	3	1	**18**
**Kennel**	0	5	0	0	**5**
**Nettle**	0	4	1	0	**5**
**Old Oak**	0	10	2	4	**16**
**Septic Tank**	0	3	1	1	**5**
**Top**	0	9	1	0	**10**
**West**	6	7	2	0	**15**
**Woodrush**	0	6	1	2	**9**
**Wych Elm**	0	5	0	0	**5**
**Yew**	0	10	2	3	**15**
**Total**	**14**	**84**	**18**	**12**	**128**

Table S2

Analytical specificity of RD4 assay tested on 100 Mycobacteria species and 21 other bacteria species. Signal was detected only from *M. bovis* AF2122/97, *M. bovis* BCG Pasture and *M. bovis* type strains from 10 x 10 panel of most common UK spoligotypes.

Species	Number of strains tested
Mycobacterium abscessus	2
Mycobacterium agri	1
Mycobacterium aichiense	1
Mycobacterium aquae	1
Mycobacterium asiaticum	1
Mycobacterium aurum	2
Mycobacterium avium	1
Mycobacterium canetti	1
Mycobacterium chelonae	2
Mycobacterium chitae	1
Mycobacterium chubuense	3
Mycobacterium duvalii	1
Mycobacterium flavescens	1
Mycobacterium fortuitum	7

Mycobacterium gadium	1
Mycobacterium gastri	1
Mycobacterium gilvum	2
Mycobacterium gordonae	3
Mycobacterium hiberniae	2
Mycobacterium intracellulare	1
Mycobacterium kansasii	5
Mycobacterium marianum	1
Mycobacterium marinum	1
Mycobacterium microti	3
Mycobacterium nonchromogenicum	2
Mycobacterium obuense	2
Mycobacterium peregrinum	2
Mycobacterium pinnipedii	1
Mycobacterium phlei	3
Mycobacterium rhodesiae	1
Mycobacterium scrofulaceum	1
Mycobacterium smegmatis	3
Mycobacterium szulgai	1
Mycobacterium terrae	3
Mycobacterium thermoresistibile	1
Mycobacterium tuberculosis	3
Mycobacterium ulcerans	1
Mycobacterium vaccae	2
Mycobacterium xenopi	2
Norcardia petroleophila	1
Nocardia brevicatena	1
Rhodococcus coprophilius	1
Rhodococcus leteus	1
Streptomyces plantensis	1
Streptomyces griseus	1
Streptomyces caesius	1
Streptomyces coelescens	1
Streptomyces coelicolor	6
Streptomyces cyanocolor	1
Streptomyces griseoruber	1
Streptomyces lividans	1
Streptomyces tuirus	1
Streptomyces violaceolatus	1
Streptomyces violaceoruber	1
Escherichia coli (Mach 1)	1

Table S2:

The relative odds of obtaining a positive sample per social group across the year.

Social group	Odds of a positive	Confidence Interval	P Value
**Old Oak**	1	-	-
**Yew**	1.86	0.56 – 6.18	0.31
**Kennel**	2.27	0.59 – 8.73	0.23
**Colliers Wood**	2.32	0.70 – 7.73	0.17
**Woodrush**	2.42	0.74 – 7.92	0.14
**Beech**	3.25	1.06 – 9.95	0.04
**Top**	4.03	1.33 – 12.15	0.013
**Wych Elm**	4.61	1.47 – 14.43	8.00×10^{-3}
**Honeywell**	5.24	1.73 – 15.84	3.37×10^{-3}
**Septic Tank**	6.51	2.17 – 19.56	8.43×10^{-4}
**West**	7.03	2.40 – 20.57	3.67×10^{-4}
**Nettle**	22.73	7.81 – 66.19	1.01×10^{-8}
